# Effects of bacterial inoculation on lignocellulose degradation and microbial properties during cow dung composting

**DOI:** 10.1080/21655979.2023.2185945

**Published:** 2023-07-20

**Authors:** Liuyan Zhou, Xinping Yang, Xiaowu Wang, Lei Feng, Zhifang Wang, Jinping Dai, Huitao Zhang, Yuqing Xie

**Affiliations:** a Institute of Microbiology Applications, Xinjiang Academy of Agricultural Sciences, Urumqi, Xinjiang PR China; b Xinjiang Key Laboratory of Special Environmental Microbiology, Xinjiang Academy of Agricultural Sciences, Urumqi, Xinjiang, PR China

**Keywords:** Compost, Lignocellulose degradation, Microbial communities, Transcriptome, Inoculation

## Abstract

Inoculation with exogenous microbial agents is a common method to promote organic waste degradation and improve the quality of compost. However, the biotic effects of different microbial agents are often quite different. To evaluate the potential effects of a complex bacterial agent comprised of three strains (belonging to *Bacillus* and *Geobacillus*) on lignocellulose degradation and the underlying microbial mechanisms during cow dung composting, two lab-scale composting experiments, a control and a bacterial inoculation treatment, were established. The results suggest that bacterial inoculation accelerated the rate of temperature increase and extended the thermophilic phase. Compared to those in the negative control group, cellulose, hemicellulose, and lignin degradation rates in the inoculated group increased from 53.3% to 70.0%, 50.2% to 61.3%, and 46.4% to 60.0%, respectively. The microbial community structure and diversity in the compost were clearly changed by the bacterial inoculation. Moreover, stamp analysis showed that inoculation modulated the key compost microbial functional populations linked to the degradation of lignocellulose. Correlation matrix analysis indicated that the expression of bacterial lignocellulolytic enzymes is closely related to key microbial functional populations. Overall, the results confirm the importance of bacterial inoculation, and have important implications for promoting the efficiency and quality of cow dung compost.

## HIGHLIGHTS

- The effects of three *Bacillus* and *Geobacillus* strains on compost were established.
- Adding the complex bacterial agent increased the thermophilic phase.
- Inoculation promoted the abundance of key lignocellulose-degrading microbes.
- These findings will help promote the efficiency and quality of cow dung compost.

## GRAPHICAL ABSTRACT

**Figure:**
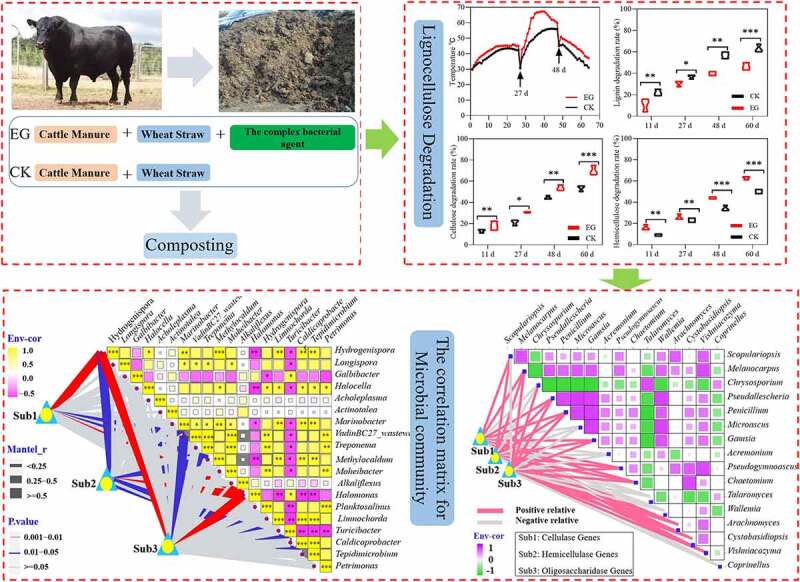

## 1. Introduction

A thriving economy fuels rapid increases in living demands and consumption. An investigation reported that the production of cattle meat and whole fresh cow milk reached 66 million tones and 675 million tones, respectively, and the total number of cows in 2017 was approximately 1492 million worldwide (National Bureau of Statistics of China 2019) [1]. Therefore, reducing the negative impacts and improving resource utilization of cow manure has become an urgent and important problem in the development of animal husbandry. Cow manure is considered a valuable source of fertilizer as it contains plentiful nutrient elements (nitrogen, phosphorus, and potassium). However, it can also cause hygienic hazards, odor, and ground water pollution from the leaching of pollutants if not properly treated. Thus, it should be handled and managed properly before being applied to farmland as an organic fertilizer.

Aerobic composting is an economical and environmentally friendly technology for treatment of organic waste [2]. This technology involves the complex biodegradation of a mixture of solid substrates conducted by a microbial community composed of various populations under aerobic conditions [3]. Raw organic materials are mineralized and stabilized into biologically stable, humic-like substances [3]. Aerobic composting is an economical and environmentally friendly technology for treatment of organic waste [2]. This technology involves the complex biodegradation of a mixture of solid substrates conducted by a microbial community composed of various populations under aerobic conditions [3]. Raw organic materials are mineralized and stabilized into biologically stable, humic-like substances [3]. Composting can also kill pathogens, such as *Escherichia coli* and *Salmonella* spp., and eliminate odor and heavy metals [4]. The finished compost product has desirable characteristics regarding odor, weed seed, and pathogens; thus, this technique has been widely used to treat organic waste in agriculture and animal husbandry [5]. Compost results from microbiological processes, and organic matter decomposition strongly relies on the activity of microorganisms [6]; therefore, maintaining a beneficial bacterial composition is important for composting. However, traditional composting typically involves a slow decomposition process. Many studies have found that the addition of key functional microorganisms to the compost is a promising strategy for extending the high-temperature duration, reducing nutrient loss, accelerating the decomposition of the compost, and promoting the maturity of materials [7–9]. With sustainable development becoming a future trend, efficient and stable microbial agents have broad application prospects for cow manure treatment.

The main organic components of cow dung are cellulose, hemicellulose, protein, fat, and lignin, which differ in biodegradability during composting [10]. Carbohydrates and crude fat are easily degraded in compost while cellulose and hemicellulose are relatively difficult to degrade, and lignin is relatively non-degradable [11]. Hence, the degradation of hemicellulose, cellulose, and lignin is an important problem in the compost fermentation process [12]. Recently, there have been many attempts to improve the efficiency and quality of compost by inoculation with degrading microbiological strains, such as *Trametes versicolor*, *Thermoactinomyces* sp., *Trichoderma harzianum*, *Rhizopus oryzae*, *Bacillus* sp., and *Bacillus stearothermophilus* [13–15] or lignocellulose-degrading and cellulose-degrading microflora [16,17]. Studies have shown that the degree of aromaticity and stability of dissolved organic matter and humic substances are substantially enhanced after inoculation of a multifunctional thermophilic microbial consortium in manure – sugarcane leaf composting [18]. Furthermore, the addition of bacteria to cattle manure compost promotes microbial activity and the degradation of cellulose-rich waste [19], and inoculation with *Phanerochaete* and *Chrysosporium* has been reported to improve the physical and chemical parameters and increase the substrate utilization rate [20]. These findings imply that applications of key microbial inoculants are critical to the composting process, which is helpful in achieving sustained benefits in composting. Most of these studies provided detailed descriptions of the composting process by analyzing temperature, microbial community dynamics, and elemental (C, N, P) transformation with the aim of finding suitable microbial agents. However, few studies have analyzed the functional aspects of key microbiota; therefore, the microbial mechanism of microbial inoculations remains unclear. In this regard, metatranscriptomics is a valuable approach to expand the repertoire of known biodegrading microorganisms and their active functional metabolic potential during composting. In the context of rapid development of the animal husbandry industry, screening for efficient and stable microbial agents and achieving diverse microbial communities in the compost plays a vital role in improving compost quality and efficiency.

In the present study, we investigated the effects of a complex bacterial agent (three strains belonging to *Bacillus* and *Geobacillus*) on lignocellulose degradation and microbial properties during cow dung composting. In addition, we tracked the dynamics of environmental factors and the succession of the bacterial and fungal community composition during composting. The potential links between lignocellulose-metabolizing enzymes and the specific microbial groups were then established by a correlation matrix. Our study is expected to provide new insights into the role and adjustability of the microbiome of compost, and provide clues for more accurate control of the composting process and development of more efficient and stable microbial agents.

## 2. Materials and methods

### 2.1. Materials and inoculum

Cow dung compost was collected from a cattle breeding farm located in Xishan, Xinjiang Uygur Autonomous Region, China (43°81’30‘′N 87°57’45‘′E) in August 2018. As the major carbon substrate, air-dried reed straw was shredded into pieces less than 5 cm long and mixed with the cow dung (1:3, v/v). The two treatment groups were as follows: cattle manure + wheat straw (negative control, CK) and cattle manure + wheat straw + compound microbial inoculant (inoculated group, EG). Each treatment was performed in triplicate. Each pile was approximately 1.0 m high, 0.8 m wide, and 1.2 m long with a moisture content of 60% outdoors. The compost was monitored for 70 days, and the piles were turned on days 27 and 48 using a forklift. The physicochemical properties of the substrates are listed in Table 1. No ethics approval was required for this study as it involved no human participants or animals

**Table 1.** Characteristics of raw materials used in the bacteria-inoculated treatment and control groups in this study.

Parameters	pH	C/N (%)	TOC (%)	OM (%)	Moisture (%)	Carbon Source	Weight (kg)
Treated	9.2	30.0	28.3	50.1	60.0	Straw	1000.0
Control	9.2	30.0	28.3	50.0	60.0	Straw	1000.0

Abbreviations: OM, organic matter; TOC, total organic carbon (TOC=OM/1.724); C/N, the ratio of total organic carbon to total Kjeldahl nitrogen.

Three different test strains were used in the compound microbial inoculant (0.1%, co-inoculation, 1:1:1 v/v; OD_600_ = 0.6): (i) Q3, *Bacillus subtilis*NCIB 3610^T^100%; (ii) NM6, *Geobacillus thermoleovorans* KCTC 3570^T^ 100%; and (iii) ND, *Bacillus cereus* ATCC 14,579^T^ 100% (Table S1), which were separated from compost samples based on the activity of cellulases, amylases, lipases, and proteases in our previous study [21]. They are mesophilic or thermophilic bacteria, and were cultured in NF9 liquid medium (sucrose, 20.0 g/L; peptone, 2.0 g/L; corn syrup, 5.0 mL/L; and Na_2_PO_4_, 1.0 g/L). Although the growth of strains Q3 and ND would be restrained to some extent, they can endure temperatures above 50°C. The total proportions and microbial activity of the three different test strains were determined in our preliminary experimental research. Additionally, single bacteria from the three different test strains were used in composting during the preliminary experimental research. However, the results showed that the effect of single application was lower than that of mixed application in cellulose degradation, temperature, and seed germination rate. Therefore, in this study, we concentrated on the influence mechanism of mixed microbial agents on the microbial community during cow dung composting.

### 2.2. Sample collection and physicochemical parameter analysis

Before turning the compost pile, samples were collected simultaneously from the upper, central, and lower layers of the treatment groups on days 0 (initial phase), 11 (mesophilic phase), 27 (mesophilic phase), and 48 (thermophilic phase). To obtain homogenized samples, the subsamples were blended as a representative sample of each group at each time point. All representative samples (approximately 1.0 kg per sample) were divided into two groups: one was stored at −80°C for bacterial and fungal DNA extraction and the other was air-dried to determine the physicochemical environmental factors. The average temperature (T) was obtained by measuring the temperature at the surface, core, and bottom of the piles using a portable thermometer. The electrical conductivity (EC) and pH were measured after shaking equilibration at a 1:10 ratio of the wet weight of the representative sample to distilled water. The air-dried samples were heated at 550°C for the determination of organic matter content (OM) [22]. Furthermore, the germination index (GI) and hemicellulose, cellulose, and lignin contents were determined according to Ouyang et al. [23] and Van Soest et al. [24].

### 2.3. DNA extraction and high-throughput sequencing

A FastDNA spin kit for soil (MP Biomedicals, Irvine, CA, USA) was used to extract total DNA from the 24 compost samples, according to the manufacturer’s protocols. Diversity analysis of the bacterial and fungal communities was performed by Allwegene Tech Co., Ltd (Beijing, China) using the Illumina MiSeq platform (Illumina, San Diego, CA, USA). For bacteria, the V3-V4 region of the 16S ribosomal RNA (rRNA) gene was amplified using two primers (338F, 5’-ACTCCTACGGGAGGCAGCAG-3;“ 806 R, 5”-GGACTACHVGGGTWTCTAAT-3“) [25]. For fungi, the primers ITS1F (5”-CTTGGTCATTTAGAGGAAGTAA-3“) and ITS2R (5”-GCTGCGTTCTTCATCGATGC-3’) were used to amplify the internal transcribed spacer (ITS) region [26]. QIIME (1.9.1) was used to filter the quality of the raw sequence data. Subsequently, the clean sequences were assigned to operational taxonomic units (OTUs, 97.0% similarity) using UPARSE [27] in the SILVA database (for bacterial 16S rRNA gene) and the UNITE database (for fungal ITS rDNA) for the sequence reference set. The 16S rRNA and ITS gene sequences obtained in this study have been deposited in the NCBI Sequence Read Archive under accession numbers PRJNA772265 and PRJNA772727, respectively.

### 2.4. RNA extraction and transcriptome analysis

Transcriptome sequencing and analysis were performed by Allwegene Tech Co. Ltd. (Beijing, China). Samples were collected from the EG group on days 0 (initial phase) and 48 (thermophilic phase), with three biological replicates for RNA extraction. Paired-end reads were generated after library preparation and Illumina sequencing. PandaSeq was used to join the paired-end sequences [28] and low-quality reads were filtered using Trimmomatic [29]. Subsequently, sequencing data analysis and differentially expressed gene (DEG) estimation, including quality control and comparative analysis, were performed. Quality control of the sequencing data was conducted using FASTA QC, and filtered reads were considered for further analysis. Finally, transcription factors, including Gene Ontology (GO) enrichment and Kyoto Encyclopedia of Genes and Genomes (KEGG) pathway enrichment, were used for annotation and pathway analysis. The expression of each unigene was estimated by fragments per kilobase of transcript per million fragments mapped read values. Genes with a threshold of fold change >2.0 (or <0.5) and a *P*-value <0.01 were set as the threshold for significant DEGs. The transcriptomic raw sequencing data were submitted to NCBI under BioProject ID PRJNA772758.

### 2.5. Statistical analyses

Prism GraphPad version 8.0 (GraphPad Software, San Diego, CA, USA) was used for tabulating and processing related data. The difference in microbial community composition between the two different treatments was evaluated with principal component analysis (PCA) and permutational multivariate analysis of variance based on both unweighted and weighted UniFrac distances using QIIME 2.0 [30]. The distribution of the bacterial and fungal communities at the phylum level was demonstrated using Circos diagrams (https://www.bioincloud.tech). The significance level was defined as *P* < 0.05, based on analysis using SPSS, Version 23.0 (IBM Corp., Armonk, NY, USA). The correlation matrix was analyzed using RStudio software (RStudio, Boston, MA, USA).

## 3. Results and discussion

Currently, composting is one of the most effective methods for treating fecal waste on large-scale livestock and poultry farms. The aim of this study was to explore the potential effects of bacterial inoculation (combined bacterial agent using three strains belonging to *Bacillus* and *Geobacillus*) on lignocellulose degradation and the underlying microbial mechanisms during cow dung composting. First, we found that bacterial inoculation considerable accelerated the rate at which the temperature increased and enhanced lignocellulose degradation. Second, the data analysis showed that bacterial inoculation improved the richness and diversity of the composting ecosystem and the key compost microbial functional populations (genus level). Finally, the expression levels of lignocellulose were analyzed, and the correlation matrix among carbohydrate-active enzyme database (CAZY) family genes and the differences in the microbial communities in the EG group were established.

### 3.1. Changes in physicochemical parameters during composting

**Figure 1.**
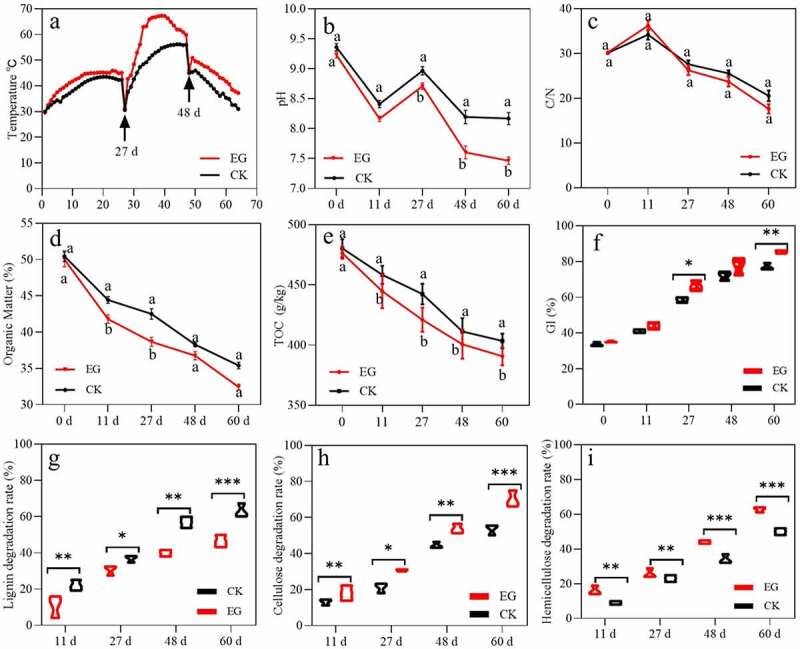
Changes in physicochemical parameters during the composting process of treated and control groups.

During the mesophilic phase, the temperature of the piles increased quickly and steadily, and the temperature of the EG group was slightly warmer than that of the CK group (Figure 1a). After turning the piles over (day 27), the temperature increased rapidly again and subsequently entered the thermophilic stage (>50°C). Although the temperature trends of the two treatment groups were similar, the temperature in the EG group was substantial higher than that in the CK group. The EG group reached the highest temperature on day 35 at 67.3°C and remained above 50.0°C for 27 days. In contrast, the CK group reached the highest temperature on day 44 at 53.4°C and remained above 50.0°C for 8 days. Subsequently, the temperature of the two treatment groups dropped on days 43 and 45, respectively, and entered the cooling stage. These results indicate that EG group likely possessed more available functional microorganisms, leading to more organic waste being metabolized and releasing more energy. An appropriately high temperature and a longer thermophilic phase not only kill pathogens and weed seeds but also increase the safety of the compost product [31].

(a) Temperature, (b) pH, (c) C/N, (d) OM, (e) TOC, (f) germination index (GI), (g) lignin degradation rate, (h) cellulose degradation rate, and (i) hemicellulose degradation rate. All data are the mean of three replicates and error bars indicate standard deviations. Different letters (a, b) indicate significantly differences at the *P* < 0.05 level.

The pH of the two treatments first decreased and then increased with composting time (Figure 1b). The reason for the pH decrease may be due to the microorganisms rapidly decomposing large quantities of easily degradable organic matter that caused organic acids to be produced [32] and simultaneously consumed some nitrate-nitrogen [33]. The pH values of the two treatment groups decreased after entering the thermophilic phase and were significantly lower in the EG group than those in the CK group. High temperatures generally cause NH^4+^-N to volatilize to NH_3_, while thermophilic microorganisms decompose organic matter and produce small molecule acids. During the cooling-off period, the pH of two treatments slowly decreased and kept approximately 7.5–8.5 until the end of compost because of the ammonia emission and ammonium oxidation by nitrobacteria [20]. Wang et al. [34] pointed out that the optimal pH value of final composting production ranged from 6.9 to 8.3. The EC of the two treatment groups was above 4.0 mS·cm^−1^ throughout the composting process (Figure S1) and appeared to be related to the local characteristics of the soil environment [35]. This may prove problematic, as a high soluble salt content inhibits crop growth. Moreover, the content of available nitrogen in the EG group was higher than that in the CK group; microbial inoculation may have reduced the volatilization of ammonia and increased the available nitrogen content.

It is widely accepted that variation in the ratio of total organic carbon to total Kjeldahl nitrogen (C/N) can reflect the stability and maturity of compost [36]. As presented in Figure 1c, the C/N ratio reached a maximum value on day 11, and then rapidly declined and became flat. The increase in the C/N ratio at day 11 might be related to the presence of lignocellulose, which was difficult to degrade in the raw materials. After 11 days of composting, the C/N values decreased until the end of composting, which is consistent with the mineralization of organic substances [37]. It has been demonstrated that compost can be considered mature when the C/N value is below 20 [36]. When the composting was complete, the C/N was 20.5 in the CK group and 17.6 in the EG group, indicating that the addition of microbial inoculants had achieved maturity. This result can likely be ascribed to the optimum temperature and pH benefitting the decomposition of organic substances and nitrogen conservation.

Figure 1d illustrated the change of organic matter content (OM) throughout the composting period. The highest values of OM content were appeared at initial phase, which were similar nearly 50.0% in two treatments. The OM content decreased in two treatments during the first 11 days, probably due to the availability of easily degradable OM such as carbohydrates, protein, and fats, etc., which could be utilized as energy source by microorganism [38]. Compared to CK group, the OM content of EG group declined sharply at mesophilic and thermophilic phase. This result might be due to addition of microbial inoculants, which could improve microbial activity and increase the degradation of OM. At the end of experiment, the content of OM declined to 35.4% and 32.4% at CK and EG, respectively. The OM content of inoculation group was significantly lower than that in CK on day 60 (*P* < 0.05). The above results indicated that addition of microbial inoculants showed better effect on enhancing the degradation rate of OM.

### 3.2. Evolution of lignocellulose fractions during composting

The activity of lignocellulose decomposition depends on the species and abundance of lignocellulose-degrading microorganisms in the mixture. The cellulose, hemicellulose, and lignin contents of the two treatment groups gradually decreased as composting progressed (Figure 1g-i). The EG group produced significantly higher cellulose, hemicellulose, and lignin degradation rates (*P* < 0.05). Specifically, the cellulose, hemicellulose, and lignin degradation rates in the EG group increased from 53.3% to 70.0%, 50.2% to 61.3%, and 46.4% to 58.9%, respectively, in nearly finished compost (day 60) compared to those in the CK group.

Degradation of cellulose and lignin (35.2% and 32.9%, respectively) occurred in the two treatment groups during the thermophilic phase, which was consistent with previous reports [39,40]. Xiao et al. [41] found that cellulose degradation is faster during the high-temperature phase. The EG group generated a higher temperature and maintained a longer thermophilic period, which improved the decomposition efficiency of cellulose and lignin. In addition, cellulose was tightly cross-linked with lignin in the lignocellulose matrix; therefore, the internal lignin components were more exposed when a considerable amount of cellulose was removed [42]. Therefore, it promoted more effective performance of lignocellulolytic enzymes secreted by the microorganisms in the EG group compared to those in the CK group.

### 3.3. Seed germination index (GI) analysis

The seed GI has been used to assess compost maturity quickly and efficiently and has been widely accepted by researchers [43]. Composts with a GI value >80.0% are considered mature according to Bernal et al. [36]. The GI was analyzed using the aqueous extracts of fresh Chinese cabbage. The GI of the EG and CK groups increased gradually during the composting process and eventually increased from 33.6% and 35.1% on day 0 to 90.7% and 83.0% on day 60, respectively (Figure 1f). This increase in the GI mostly resulted from the decomposition of toxic materials. The results indicated that the microbial agent inoculation resulted in faster maturity and lower plant toxicity of the compost. A reasonable explanation for this phenomenon was that inoculation could increase the population of microorganisms, which accelerated the decomposition rate of organic matter and contributed more heat and short chain volatile fatty acids. Despite the existence of short chain volatile fatty acids and the large quantity of NH_4_^+^-N from microbial ammonization, which might be phytotoxic to the growth of plants [44,45], an increased number of microorganisms and higher temperature accelerates the decomposition of phytotoxic substances such as short chain volatile fatty acids and NH_3_ emissions [46]. The addition of microbial inoculants had a substantial impact on the GI of the final compost product, and the final products of the EG group were mature and non-phytotoxic, which is in line with the national standard requirement [47].

### 3.4. Changes in bacterial and fungal community diversity during composting

**Figure 2.**
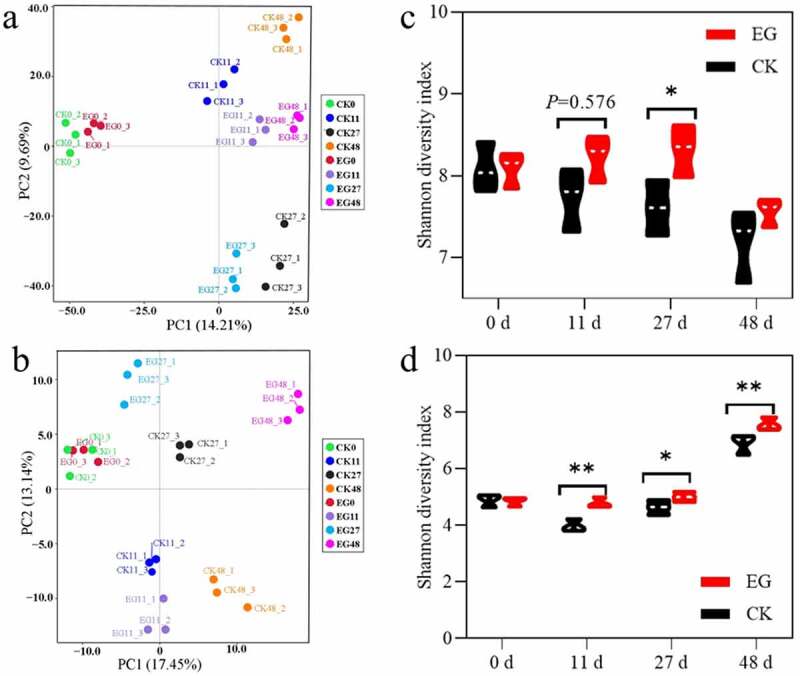
Succession of the microbial community during the composting process of treated and control groups. Principal component analysis (PCA) and *α*-diversity based on Shannon indices of bacterial (a, b) and fungal (c, d) community composition in treated and control groups (*n* = 3 for each group). Boxes are vertically bounded by the first and third numerical value and the center line represents the median.

After the quality ﬁlter and removal of potential chimeras, a total of 3,799,998 high-quality bacterial and 2,111,338 fungal sequences were generated for 48 samples across the two treatment groups. Regarding the *β*-diversity, all compost bacterial or fungal samples did not cluster together according to the composting stage. The distance of samples between different treatments obviously showed that the treatment in the EG group significantly affected the fungal and bacterial communities, especially during the cooling and maturity stages Figure 2(a-c). At the initial thermophilic stage, the rapid increase in temperature was the most predominant environmental factor that drove thermophilic microorganisms to replace mesophilic microorganisms, leading to huge changes in the microbial community composition. Additionally, the microbial community in the compost was found to be significantly affected by raw materials and other environmental factors besides temperature (e.g. moisture, C/N, and water-soluble organic carbon). The Shannon diversity indices were generally different between the compost samples in the EG and CK groups Figure 2b-d. For bacteria and fungi, the EG samples showed significantly higher values for all diversity indices than those for the CK samples (Figure S2). Although the *α*-diversity of the EG group was relatively more stable from the initial to the mesophilic period (day 27), it changed significantly in both the EG and CK groups during the thermophilic period. The Shannon index of fungi and bacteria did not show a uniform trend between the two treatments, indicating that fungal and bacterial diversity are susceptible to environmental factors (e.g. temperature, moisture, C/N, and water-soluble organic carbon). Overall, the richness and diversity of the EG group was higher than those in the CK group during the composting process, indicating that inoculating with exogenous microbial agents improved the richness and diversity of the composting ecosystem. The Venn diagram shows that the EG and CK samples possessed 1,214 (8.1%) and 933 (6.0%) bacterial OTUs shared by the four different compost stages, respectively (Figure S3). Furthermore, 64 (4.4%) and 52 (3.5%) fungal OTUs were shared by the four compost stages, respectively. The results suggested that EG slightly enhanced the core bacterial OTUs but not the core fungal OTUs.

**Figure 3.**
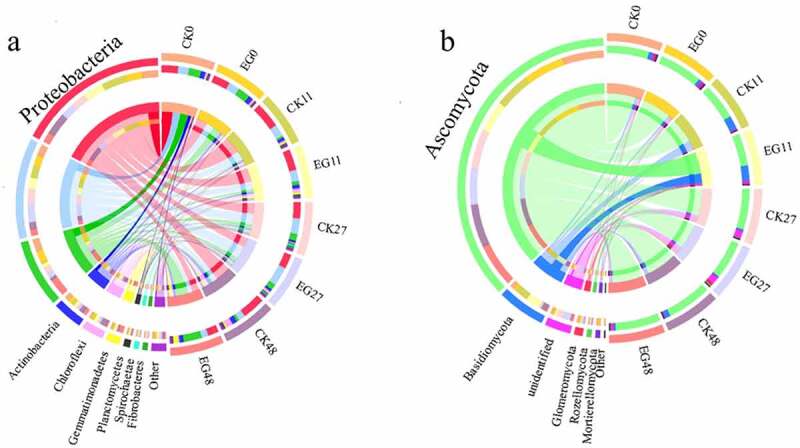
Bacterial (a) and fungal (b) community composition at the phylum level during composting.

At the phylum level, the top nine phyla represented 95–97% of the total bacterial community, including Proteobacteria (21.8–52.4%), Bacteroidetes (14.5–39.0%), Firmicutes (4.0–37.3%), and Actinobacteria (4.2–9.6%). The remainder belonged to the phyla Spirochaetae, Chloroflexi, Gemmatimonadetes, Planctomycetes, and Fibrobacteres (Figure 3a). Owing to the copiotrophic strategies of Proteobacteria and Bacteroidetes, they usually show a rapid growth response to resource availability [48]. Thus, Proteobacteria and Bacteroidetes initially increased and then decreased during composting. In contrast, Firmicutes increased significantly in the thermophilic phase and were able to secrete various extracellular thermostable enzymes and degrade some macromolecular substrates, such as protein, pectin, and cellulose. The addition of microbial inoculants had no significant impact on the abundance of Actinobacteria, but Bacteroidetes was impacted by day 27. On day 48, Firmicutes and Gemmatimonadetes were more abundant in the EG group than in the CK group. Previous findings indicate that these are important bacteria for anaerobic fermentation, decomposing organic matter to hydrogen or acetic acid [49], which also matches with the levels of cellulose and lignin degradation during the composting process.

Ascomycota was the most well-represented fungal division during the composting process, comprising more than 87% of the fungal species in the thermophilic stage, and the remainder (10.0–20.0%) belonged to the phyla Basidiomycota, Glomeromycota, and Mortierellomycota (1.9–29.6%, 0.1–2.6%, and 0.3–2.7%, respectively) (Figure 3b). Ascomycota and Glomeromycota in the two treatment groups were slightly different on days 11 and 27, whereas Basidiomycota exhibited a greater difference on days 11 and 48. At a finer taxonomic level, the abundance of 13 bacterial and seven fungal taxa at the family level showed significant changes in different compost periods, among which Trichocomaceae, Glomeraceae, Anaerolineaceae, Rhodothermaceae, Limnochordaceae, and Marinilabiaceae were the most representative (Figure S4).

### 3.5. Specific differences in the microbiome during composting

**Figure 4.**
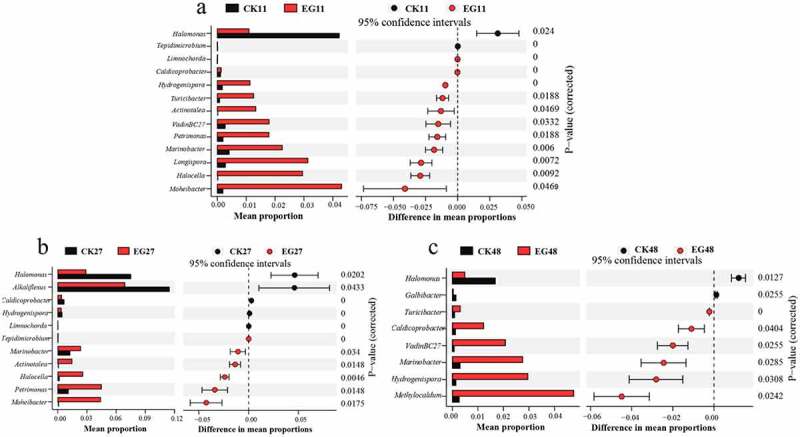
Significantly different groups (*P* < 0.05) in the bacterial community at the genus level. Corrected *P*-values were calculated using false discovery rate correction. The different colors that are represented at varying proportions in the community indicate a positive or negative difference between proportions.

The differences in taxonomic identity and abundance of bacterial and fungal taxa at the genus level were explored because of the observed differences in *α-* and *β*-diversity between the EG and CK groups. For bacteria, the genera *Moheibacter*, *Halocella*, *Marinobacter*, *Petrimonas*, and *Actinotalea* were consistently enriched in the EG group, while *Halomonas* was more abundant in the CK group on days 11 and 27 (Welch’s *t*-test, *P <* 0.05, false discovery rate [FDR]-corrected, Figure 4(a,b)). At 48 days, the genera *Methylocaldum*, *Marinobacter*, *VadinBC27_wastewater_sludge*, *Caldicoprobacter*, *Turicibacter*, and *Hydrogenispora* were more abundant in the EG group, while *Halomonas* and *Galbibacter* were more abundant in the CK group (Welch’s *t*-test, *P <* 0.05, FDR-corrected, Figure 4c). Notably, the genera exclusive to the EG and CK groups were *Marinobacter* and *Halomonas*, respectively, from day 11–48.

The *Marinobacter* genus is mostly facultative aerobic, heterotrophic, and halotolerant bacteria, and the main factor favoring *Marinobacter* abundance is hydrocarbon amendment [50]. The genus *Halomonas* is characteristically halophilic or halotolerant with a denitrification function and can secrete various metabolites (polyhydroxyalkanoates) with a basic carbon source [51]. The genera *Petrimonas*, *Actinotalea*, and *Halocella* are mesophilic and can utilize extremely tough substances, such as cellulose. In contrast, *Hydrogenispora*, *Caldicoprobacter*, and *VadinBC27_wastewater_sludge* are thermophilic biomass-degrading bacteria that can utilize complex organic compounds (chitin, xylan, and lignin) [52–54]. In terms of metabolic characteristics, they might aid in the degradation of large molecular substances and refractory organic compounds, and their abundance is strongly modulated by the pile environment and affected by temperature. Further experimentation is needed to decipher the impact of these ‘enriched’ microbes on the efficiency and quality of cow dung compost. Moreover, *Methylocaldum* was more enriched in the EG group than in the CK group (48 d; 0.11% and 3.87%, respectively), and could utilize methane, which possibly contributed to reducing methane emissions during composting [55].

**Figure 5.**
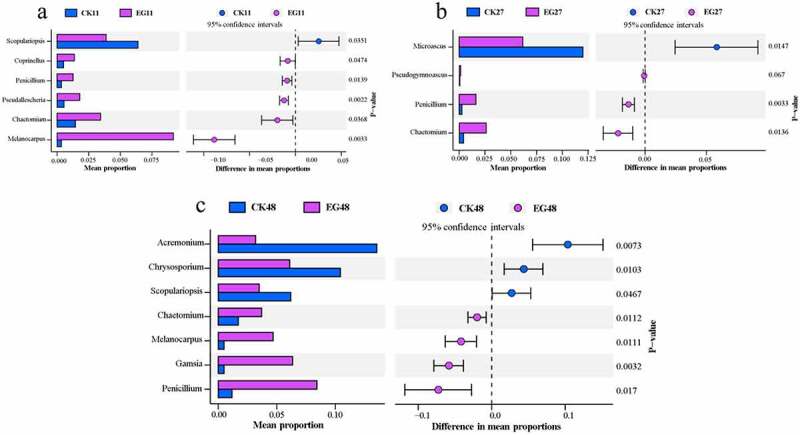
Significantly different groups (*P* < 0.05) in the fungal community at the genus level. Corrected *P*-values were calculated using false discovery rate correction. The different colors that are represented at varying proportions in the community indicate a positive or negative difference between proportions.

For fungi, on day 11, the genera *Pseudallescheria*, *Melanocarpus*, *Chaetomium*, *Coprinellus*, and *Penicillium* were more abundant in the EG group than in the CK group, whereas *Scopulariopsis* was significantly more abundant in the CK group (Welch’s *t*-test, *P <* 0.05, FDR-corrected, Figure 5a). On day 27, *Chaetomium* and *Penicillium* were enriched in the EG group; however, *Microascus* was enriched in the CK group (Welch’s *t*-test, *P <* 0.05, FDR-corrected, Figure 5b). On day 48, *Gamsia*, *Melanocarpus*, *Chaetomium*, and *Penicillium* were significantly more abundant in the EG group, whereas *Chrysosporium*, *Scopulariopsis*, and *Acremonium* were enriched in the CK group (Welch’s *t*-test, *P <* 0.05, FDR-corrected, Figure 5c). *Chaetomium* and *Penicillium* are moderately thermophilic and known for their cellulose-degrading capabilities. *Melanocarpus* are very diverse and cosmopolitan fungi, and play an important role in decomposing organic material [56,57]. They are associated with their ability to decompose complex carbohydrates, thereby contributing to carbon cycling in cow dung compost. *Microascus*, *Acremonium*, and *Scopulariopsis* were more enriched in the CK group, which could cause widespread infection [49]. Taken together, the comparative results verified that microbial inoculation modulated the abundance of specific functional groups and reduced bacterial pathogens in cow dung compost. The detailed genus levels of the microbial community are shown in Figure S5.

### 3.6. Transcriptional analysis

Transcriptomic analysis of the bacterial community in the EG group (days 0 and 48) was performed to investigate the genes encoding carbohydrate-active enzymes related to the decomposition of cow dung lignocellulose. From six samples (three biological replicates for each period), a total of 2.2 billion cleaned reads (32.2 Gb) were obtained after filtering, and each sample contained approximately 4.1–6.7 Gb (Table S2). The error rate of transcript data was 2.1–2.4%, and the Q20 and Q30 values exceeded 97.6% and 92.9%, respectively, and met the basic requirements of gene discovery. A non-redundant transcript cluster was obtained, including 997,517 unique genes with an average length of 925 bp, N50 of 1,176 bp, and N90 of 414 bp. The results indicated that 118,611 genes were upregulated and 186,660 genes were downregulated.

**Figure 6.**
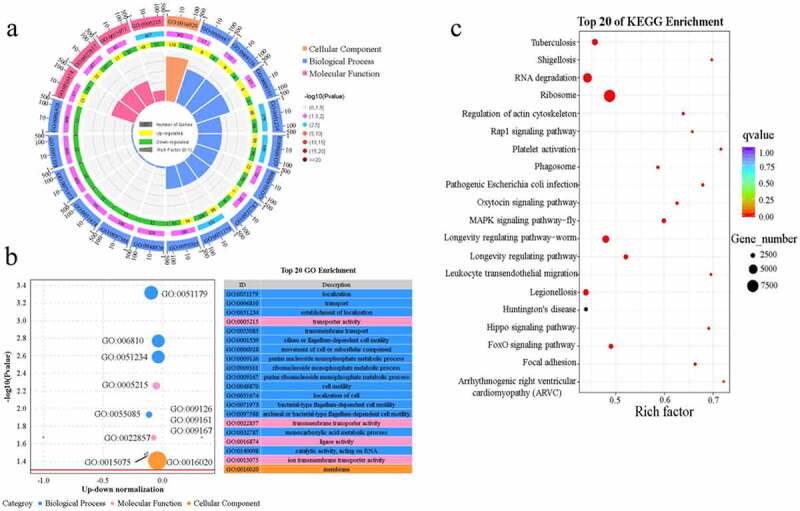
Gene Ontology (GO) and Kyoto Encyclopedia of Genes and Genomes (KEGG) analysis of differentially expressed genes (DEGs) for metabolism pathways of the bacterial community in the treated group. (a) Top 20 GO enrichment circles of DEGs, (b) GO summary graphs, and (c) KEGG enrichment analysis. There are four circles from outside to inside in (a). The first ring indicates the top 20 GO terms, and the number of the genes corresponds to the outer ring. The second ring indicates the number of genes in the genome background and *P*-values for enrichment of the upregulated genes for the specified biological process. The third ring indicates the ratio of the upregulated genes (yellow and purple) and downregulated genes (green and purple). The fourth ring indicates the enrichment factor of each GO term.

In total, 230,188 DEGs were subjected to enrichment analysis of GO functions and KEGG pathways. GO function analysis showed that 366, 316, and 423 categories were enriched in biological processes, molecular functions, and cellular components, respectively. The top 20 GO enrichment circles and summary graphs of the DEGs are presented in Figure 6 a and b. Among the top 20 enriched GO entries, the membrane related to cell components possessed the highest number of enriched factors. Fifteen items were enriched in biological processes; among them, GO: 0051179 (localization), GO: 0006810 (transport), and GO: 0051234 (establishment of localization) were enriched in more genes (194, 182, and 182, respectively), and the downregulated genes were enriched to a higher degree. Finally, four entries were enriched in molecular functions, among which the *P*-values of GO: 0015075 (transporter activity), GO: 0022857 (transmembrane transporter activity), and GO: 0016874 (ligase activity) were higher. To further understand the growth status of the microbial community, DEGs were mapped to the KEGG database. In the top 20 enriched pathways, DEGs mapped to the ribosome (ko03010) occupied the largest proportion, with ‘RNA degradation (ko03018)’ and ‘Longevity regulating pathway-worm (ko04212)’ ranking second and third (Figure 6c). Combining the analysis results of the GO functions and KEGG pathway, it was evident that these pathways were more involved in translation-, localization-, membrane-, and biological processes.

Altogether, 39907 carbohydrate-active enzyme-encoding genes were detected in different families, with 18,462 potentially involved in lignocellulose degradation, including enzymes found in the auxiliary activity, glycoside hydrolase (GH), and carbohydrate esterase families. Among them, two auxiliary activity families were lignin-degrading enzymes, and 14 GH families were cellulose-degrading enzymes, with seven carbohydrate-binding module (CBM) family accessory proteins related to cellulose degradation. Moreover, 11 GH and 11 carbohydrate esterase families belonged to the hemicellulose-degrading enzyme system, and seven CBM families assisted the catalytic function of the hemicellulase system. In addition, 14 GH families belonged to the cello-oligosaccharide-degrading enzyme system and three CBM families included cello-oligosaccharide-degrading enzymes. Using cluster analysis, we confirmed that the expression levels of lignocellulosic enzymes were significantly higher during the thermophilic period than those during the initial period (Figure S6). These results implied that an inducing mechanism supporting the high expression level of lignocellulose should exist, and it might be intimately connected with the regulation of microbial inoculation on the resident microbes in cow dung compost.

### 3.7. Relationship between CAZY family genes and the microbial community

**Figure 7.**
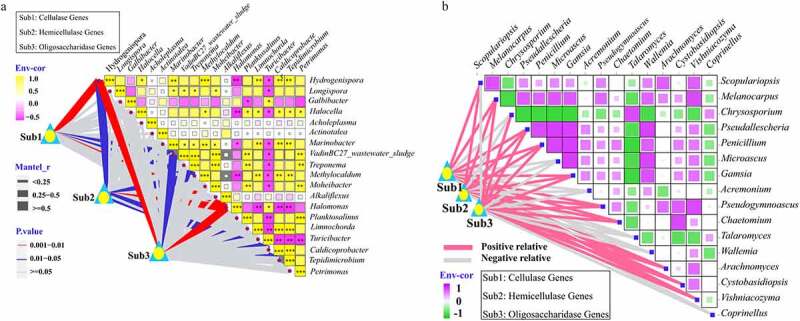
Pairwise comparisons of the microbial community at the genus level with color gradients denoting Spearman’s correlation coefficient. Lignocellulose-degrading enzyme genes were related to each other by a mantel test. (a) Bacteria; (b) fungi.

The correlation matrix among CAZY family genes and the microbial population in the EG group was explored. Complex interactions among the bacterial species were observed (Figure 7a). *Halomonas* and *Marinobacter* were exclusively present in the CK and EG groups, respectively. *Marinobacter* was significantly positively correlated (*P* < 0.05) with *VadinBC27_wastewater_sludge*, *Hydrogenispora*, *Longispora*, *Treponema*, *Methylocaldum*, *Moheibacter*, *Limnochorda*, *Caldicoprobacter*, and *Tepidimicrobium*, with the only two significant negative connections (*P* < 0.05) identified as *Halomonas* and *Turicibacter*. In contrast, *Halomonas* showed a significantly negative correlation (*P* < 0.05) with *Halocella*, *Marinobacter*, *Limnochorda*, *Turicibacter*, *Caldicoprobacter*, *Tepidimicrobium*, and *Methylocaldum*, with the only significantly positive connection identified as *Turicibacter* (*P* < 0.05). In the correlation matrix, we observed positive correlations between bacterial populations, which suggested niche overlap, as well as negative correlations, suggesting competition or amensalism [58]. The significantly enriched bacterial populations in the EG group were generally positively correlated, forming well-differentiated clusters. These significantly enriched bacterial populations consisted mainly of resident functional microbes involved in the degradation of complex organic matter.

Figure 7a shows that the expression levels of the cellulase, hemicellulase, and oligosaccharide genes were significantly related to *Hydrogenispora*, *VadinBC27_wastewater_sludge*, *Halomonas*, and *Methylocaldum* (*P* < 0.05). Figure 7b shows that the expression levels of cellulase, hemicellulase, and oligosaccharide genes were significantly related to *Chaetomium*, *Melanoleuca*, *Pseudallescheria*, *Penicillium*, *Gamsia*, *Pseudogymnoascus*, *Vishniacozyma*, and *Aspergillus* (*P* < 0.05). These functional microorganisms were more abundant in the EG group, which is an important feature of microbes associated with the degradation of lignocellulose. They might be members of the core functional microbiome and most likely better adapted to and responded to the compost environment in the EG group, such as temperature. The diversity and abundance of these microorganisms in EG, as well as their diversity in metabolic traits, make them potentially important functional microbes for compost material transformation.

## 4. Conclusion

Bacterial inoculation (combined bacterial agent containing three strains belonging to *Bacillus* and *Geobacillus*) effectively extended the thermophilic phase and enhanced lignocellulose decomposition because key microbial functional populations were regulated and controlled. The microbial community diversity and structure were clearly changed by inoculation, and the key microbial functional populations were more enriched in the EG group. In addition, there was a strong correlation between the abundance of specific functional populations and the expression levels of lignocellulose-degrading enzymes. This study has important implications for the resource utilization of livestock manure, seeking higher efficiency and quality of compost.

## Supplementary Material

Supplemental Material

## Data Availability

All data generated or analyzed during this study are included in this published article and its supplementary information files.
