# Supplementary material for: Effects of bacterial inoculation on lignocellulose degradation and microbial properties during cow dung composting
Source: Bioengineered. 2023 Jul 20;14(1):2185945. doi: 10.1080/21655979.2023.2185945 (PMC10599258; doi:10.1080/21655979.2023.2185945)
Supplement: Supplemental Material [file KBIE_A_2185945_SM7814.pdf]

Supporting Information for: Bioengineered

Effects of bacterial inoculation on lignocellulose degradation and microbial properties during cow dung composting

Liuyan Zhou[1,2], Xinping Yang[1,2], Xiaowu Wang[1,2], Lei Feng[1,2], Zhifang Wang[1,2], Jinping Dai[1,2], Huitao Zhang[1,2], and Yuqing Xie[1,2]*

[1] Institute of Microbiology Applications, Xinjiang Academy of Agricultural Sciences, Urumqi 830000, PR China

[2] Xinjiang Key Laboratory of Special Environmental Microbiology, Urumqi 830000, PR China

*Correspondence: Yuqing Xie, xyqmail@sohu.com

Running Title: Effects of bacterial inoculation during cow dung composting

Figure S1 Changes in physicochemical parameters during the composting process of treated and control groups. (a) EC, (b) Available phosphorus content, (c) Organic matter, (d) Nitrogen content. All data are the mean of three replicates and error bars indicate standard deviations.

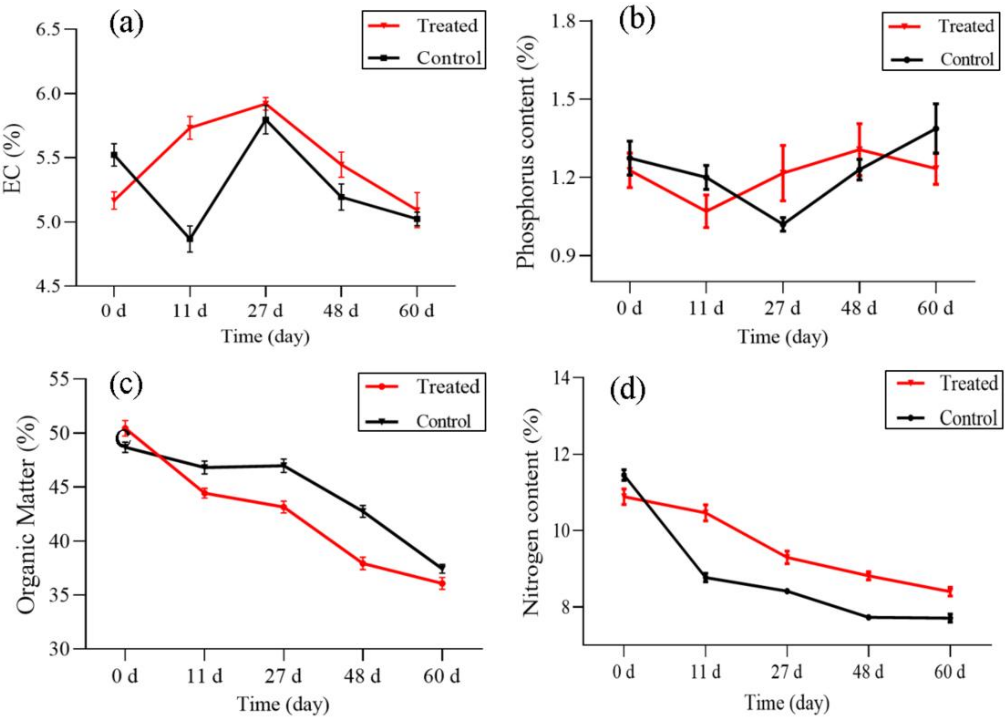

Figure S2 Alpha diversity based on Chao1 indexes of bacterial (a) and fungal (b) community composition in treated and control groups (n= 3 for each group). An OTU-based diversity analysis of bacterial (c) and fungal (d) community composition in treated and control groups (n= 3 for each group).

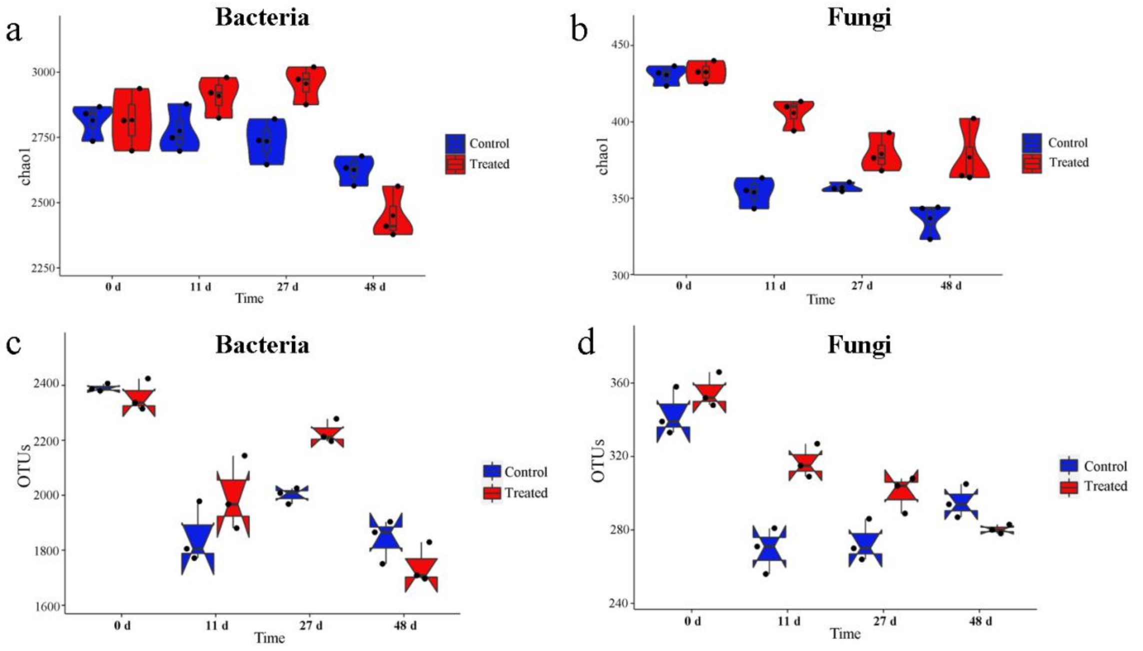

Figure S3 Venn diagram of the exclusive and shared OTUs of bacterial (a, c) and fungal (b, d) in treated and control groups among different composting periods (0 d, 11 d, 27 d and 48 d). Column chart of the exclusive OTUs of bacterial (e) and fungal (f) in treated and control groups among different composting periods.

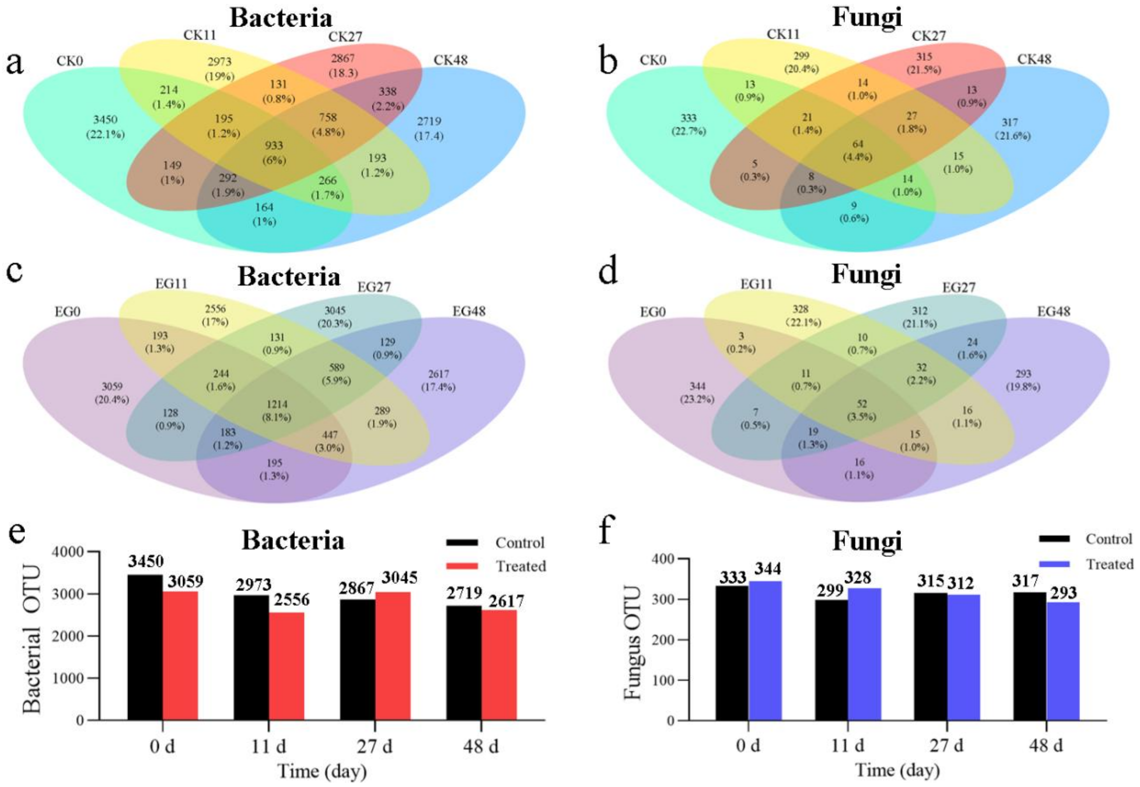

Figure S4 Changes of bacterial (a) and fungal (b) community composition in treated and control groups at the genus level (top 30) during the composting process.

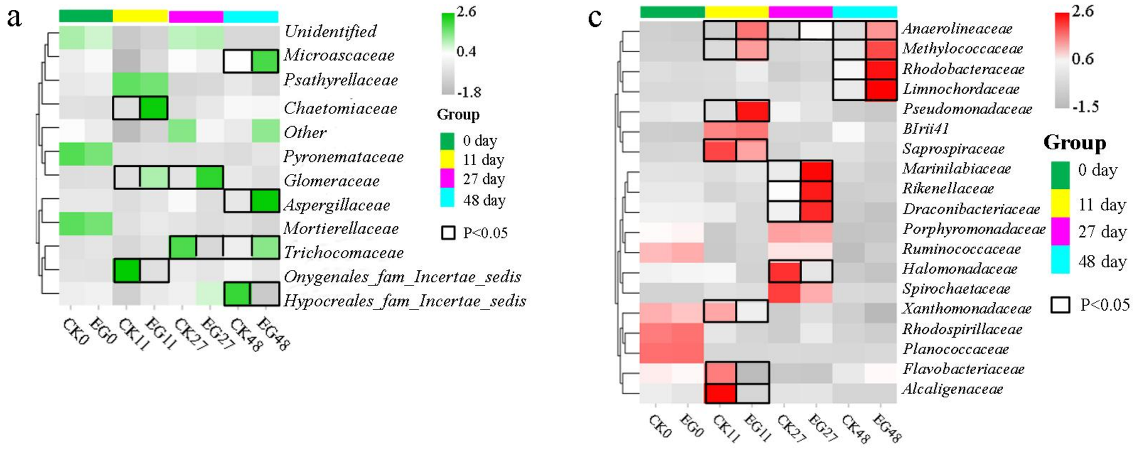

Figure S5 Changes of bacterial (a) and fungal (b) community composition in treated and control groups at the family level during the composting process. Black boxes indicate the statistical significance of differences between treatments at each time point.

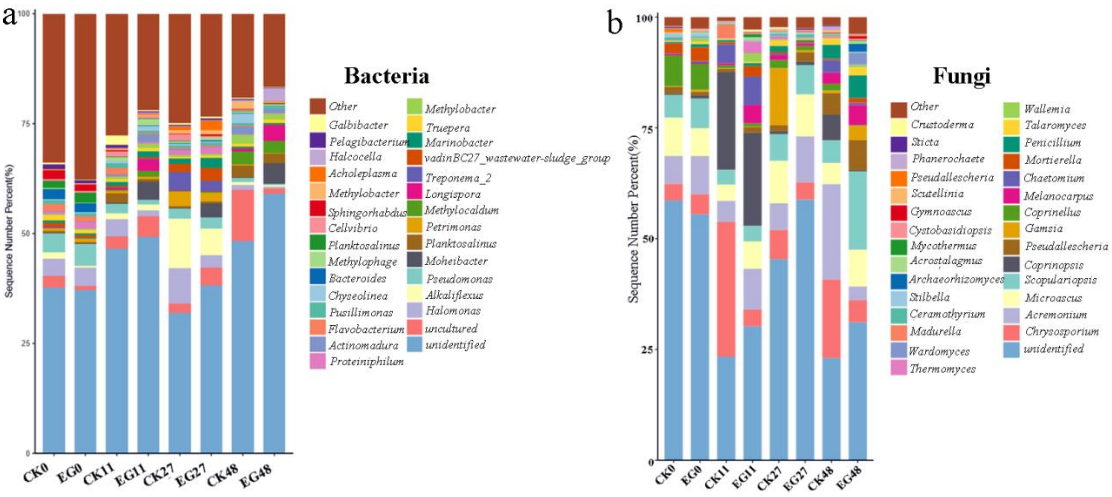

Figure S6 Expression analysis of genes encoding lignocellulosic-metabolizing on 0 and 48 days. a, Cellulose degradation; b, Hemicellulose degradation; c, Cello-oligosaccharides and Lignin degradation. GH, glycoside hydrolase; CE, carbohydrate esterase, CBM, carbohydrate-binding module, AA, auxiliary activities.

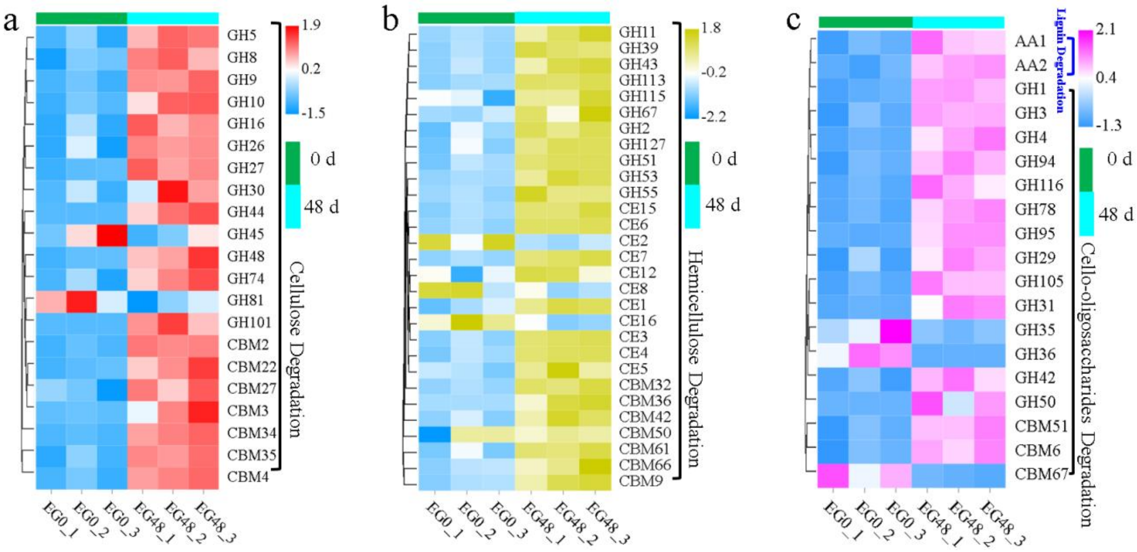

Table S1 Summary of growth temperature on properties of strains Q3, ND and NM6.

**Strain**	**Q3**	**ND**	**NM6**
16S rRNA gene sequence comparison	*Bacillus subtilis* NCIB 3610^{T} 100%	*Bacillus cereus* ATCC 14579^{T} 100%	*Geobacillus thermoleovorans* KCTC 3570^{T} 100%
Optimum (°C)	35–37	36–40	65–70
temperature range (℃)	15–56	12–58 °C	47–75

Table S2 Summary of RNA-seq reads in treatments (0 d and 48 d) groups of cow dung composting.

Sample	Raw Reads	Clean Reads	Clean Bases	Error (%)	Q20 (%)	Q30 (%)	GC Content (%)
EG0_1	29235120	29093578	4.36G	0.02	97.58	92.91	47.51
EG0_2	39726522	39531530	5.93G	0.02	97.61	93	51.28
EG0_3	27668018	27534250	4.13G	0.02	97.74	93.36	50.34
EG48_1	34478234	34282570	5.14G	0.02	97.73	93.61	67.55
EG48_2	44848784	44562490	6.68G	0.02	97.67	93.57	64.4
EG48_3	40038528	39817234	5.97G	0.02	97.88	94.06	65.73
